# Field evaluation of two novel sampling devices for collecting wild oviposition site seeking malaria vector mosquitoes: OviART gravid traps and squares of electrocuting nets

**DOI:** 10.1186/s13071-016-1557-7

**Published:** 2016-05-10

**Authors:** Sisay Dugassa, Jenny M. Lindh, Steven W. Lindsay, Ulrike Fillinger

**Affiliations:** International Centre of Insect Physiology and Ecology, Thomas Odhiambo Campus, Mbita, Kenya; School of Biological Sciences, University of Nairobi, Nairobi, Kenya; Department of Zoological Sciences, Addis Ababa University, Addis Ababa, Ethiopia; Royal Institute of Technology, Stockholm, Sweden; School of Biological and Biomedical Sciences, Durham University, Durham, UK; Disease Control Department, London School of Hygiene & Tropical Medicine, London, UK

**Keywords:** *Anopheles*, Malaria vector, Gravid trap, Electrocuting net, Semi-field, Field study

## Abstract

**Background:**

New sampling tools are needed for collecting exophilic malaria mosquitoes in sub-Saharan Africa to monitor the impact of vector control interventions. The OviART gravid trap and squares of electrocuting nets (e-nets) were recently developed under semi-field conditions for collecting oviposition site seeking *Anopheles gambiae* (*sensu stricto*) (*s.s*.). This study was designed to evaluate the efficacy of these traps for sampling malaria vectors under field conditions.

**Methods:**

Prior to field testing, two modifications to the prototype OviART gravid trap were evaluated by (i) increasing the surface area and volume of water in the artificial pond which forms part of the trap, and (ii) increasing the strength of the suction fan. Six sampling tools targeting gravid females (Box gravid trap, detergent-treated ponds, e-nets insect glue-treated ponds, sticky boards and sticky floating-acetate sheets) were compared under field conditions to evaluate their relative catching performance and to select a method for comparison with the OviART gravid trap. Finally, the trapping efficacy of the OviART gravid trap and the square of e-nets were compared with a Box gravid trap during the long rainy season in three household clusters in western Kenya.

**Results:**

The OviART gravid trap prototype’s catch size was doubled by increasing the pond size [rate ratio (RR) 1.9; 95 % confidence interval (CI) 1.1–3.4] but a stronger fan did not improve the catch. The square of e-nets performed better than the other devices, collecting three times more gravid *Anopheles* spp. than the Box gravid trap (RR 3.3; 95 % CI 1.4–7.6). The OviART gravid trap collections were comparable to those from the e-nets and 3.3 (95 % CI 1.5–7.0) times higher than the number of *An. gambiae senso lato* (*s.l*.) collected by the Box gravid trap.

**Conclusion:**

Both OviART gravid trap and squares of e-nets collected wild gravid *Anopheles gambiae* (*s.l*.) where natural habitats were within 200–400 m of the trap. Whilst the e-nets are difficult to handle and might therefore only be useful as a research device, the OviART gravid trap presents a promising new surveillance tool. Further field testing is needed in different eco-epidemiological settings to provide recommendations for its use.

## Background

Malaria control efforts have been intensified over the past decade resulting in a cut of malaria mortality in Africa by half [1]. This has been achieved by improvement in diagnoses and treatment and by scaling up vector control targeting resting and host-seeking malaria vector mosquitoes indoors. In many parts of Africa the use of long-lasting insecticidal nets has depressed the vector population entering or remaining in houses and has resulted in an increased proportion of mosquitoes biting outdoors [2–4]. However, few outdoor sampling tools [5] for malaria vectors are available which has led to increased research efforts to develop sampling tools that target outdoor populations of mosquitoes [6–11].

We recently reported the development of two novel tools for sampling gravid *Anopheles gambiae* (*s.l*.): a suction trap, known as the OviART gravid trap [12] and a square of electrocuting nets (e-nets) [13]. Both traps catch gravid females when searching for aquatic habitats to lay eggs and were developed under semi-field conditions. Here we set out to test these tools for the collection of wild mosquitoes under field conditions in western Kenya. In the first step, the prototype OviART gravid trap was modified by increasing its pond size and suction power with the aim to increase its collection efficiency, whilst five mosquito sampling devices (e-nets, detergent-treated ponds, glue-treated pond, sticky floating-transparent sheet, sticky board) were compared to the commercially available Box gravid trap under standardized field conditions to select methods for comparison with the OviART gravid trap under field conditions in a local village in a second step.

## Methods

### Study sites

**Fig. 1:**
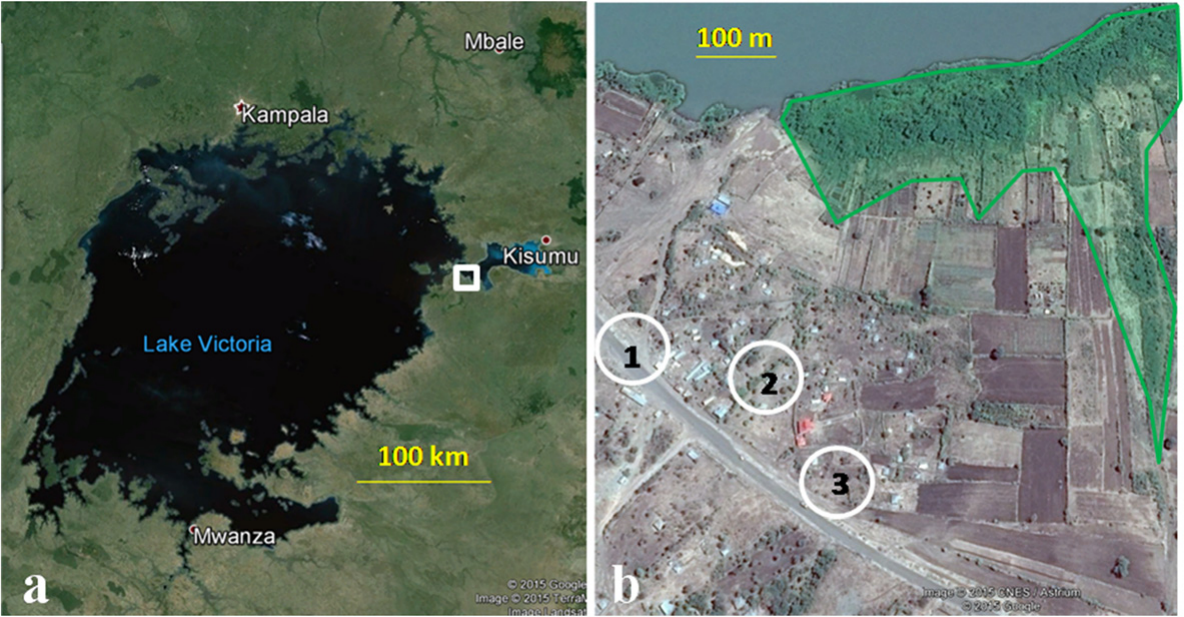
Maps of study location in western Kenya for the field evaluation of the OviART gravid trap and square of electrocuting nets. **a** Overview: white square indicates Kombe village in the Lake Victoria region in East Africa. **b** Close-up showing the location of the three household clusters (1–3 in *white circles*) and the location of nearest aquatic habitats (*green* enclosure)

**Fig. 2:**
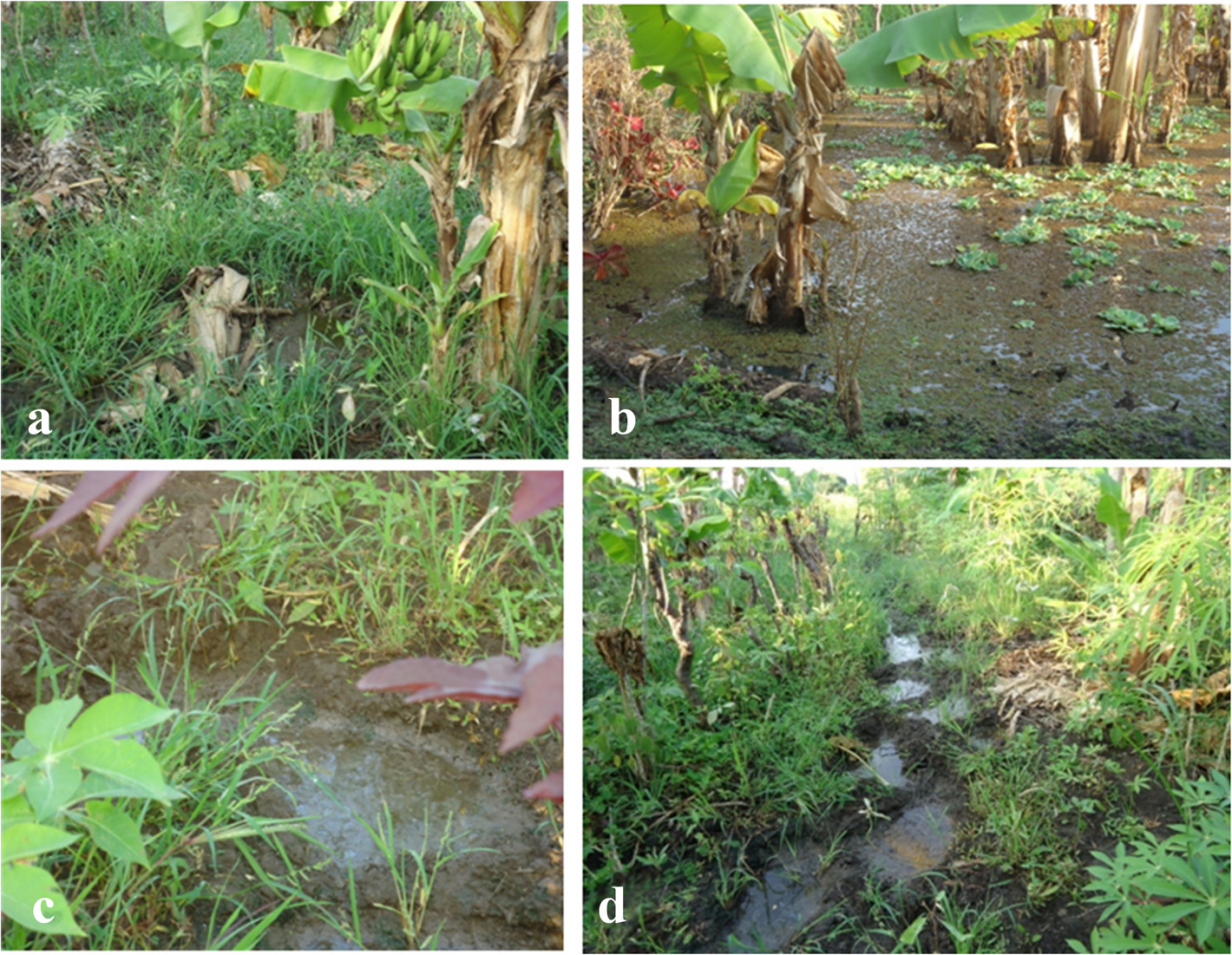
Natural aquatic habitat types found around the village field site. **a** Pit around banana plants. **b** Floodwater from Lake Victoria, covered with Azolla and Pistia. **c** Rainwater puddle. **d** Hippopotamus footprints

Semi-field experiments were done in large netting-screened greenhouses (80 m^3^) as described previously [12, 13] at the International Centre of Insect Physiology and Ecology, Thomas Odhiambo Campus (icipe-TOC), located on the shores of Lake Victoria, at Mbita [00°26′06.19″S, 34°12′53.13″E; altitude 1,137 m above sea level (a.s.l.)], western Kenya. To compare sampling efficiency of different sampling tools standardized field experiments were carried out in the agricultural fields on site of icipe-TOC. Subsequently, field work was conducted near Kombe village, approximately 2 km south of icipe-TOC (00°26′03.79″S, 34°13′02.95″E; 1,150 m a.s.l.) (Fig. 1); 300–500 m from the lake shore. Water bodies serving as potential oviposition sites for gravid malaria vectors were found 200–400 m from the trap locations. These habitats were mainly associated with agriculture in water logged areas along the lake and seasonal rivers (drains, borrow pits, freshwater marshes and footprints from hippopotami) (Fig. 2). Homesteads in this rural area usually consist of several houses and are scattered across the landscape. Houses are built of mud, or less commonly bricks with corrugated iron sheet roofing. Most houses have open eaves which allow mosquito access to the house [14]. The main income generating activities of the local inhabitants are fishing and subsistence farming.

### Mosquitoes

Insectary-reared *An. gambiae* (sensu stricto) (*s.s.*) of the Mbita strain were used for semi-field experiments. Three hundred two- to 3-day-old females and 300 males were kept in 30 × 30 × 30 cm netting cages and provided with 6 % glucose solution ad libitum at 25–28 °C and a relative humidity of 68–75 %. Water saturated cotton towels (50 × 25 cm) were folded and placed over the cages to avoid mosquito desiccation. Mosquitoes were starved of sugar for seven hours and then allowed to feed on a human arm for 15 min at 19.00 h on the same day. The same procedure was repeated 24 h later. After the first blood meal unfed mosquitoes were removed from the cages. Fed mosquitoes were kept together with males for two more days after the second blood meal before they were used in this experiment (i.e. 4–5 days after first blood meal). A small proportion of these mosquitoes might not have been gravid because most females need two blood meals to reach full gravidity and some never reach full gravidity even after three feeds. Whilst we provided two meals, we cannot guarantee that two meals were taken by all females.

### Experimental procedures

#### Modifications of the prototype OviART gravid trap

The OviART gravid trap is an odour-baited trap; the attractant is a bowl of lake water and gravid mosquitoes approaching the trap are sucked into a collection chamber by a fan powered by a battery. Two choice experiments were conducted in a semi-field system [13, 15] with treatments (prototype trap versus modified trap) positioned in diagonal corners approximately 12 m apart, 1.5 m from the two adjacent walls at a corner of the greenhouse. Two hundred gravid females were released in the centre of the semi-field system 6 m from each trap. Lake water pumped from Lake Victoria was used as oviposition medium.

**Fig. 3:**
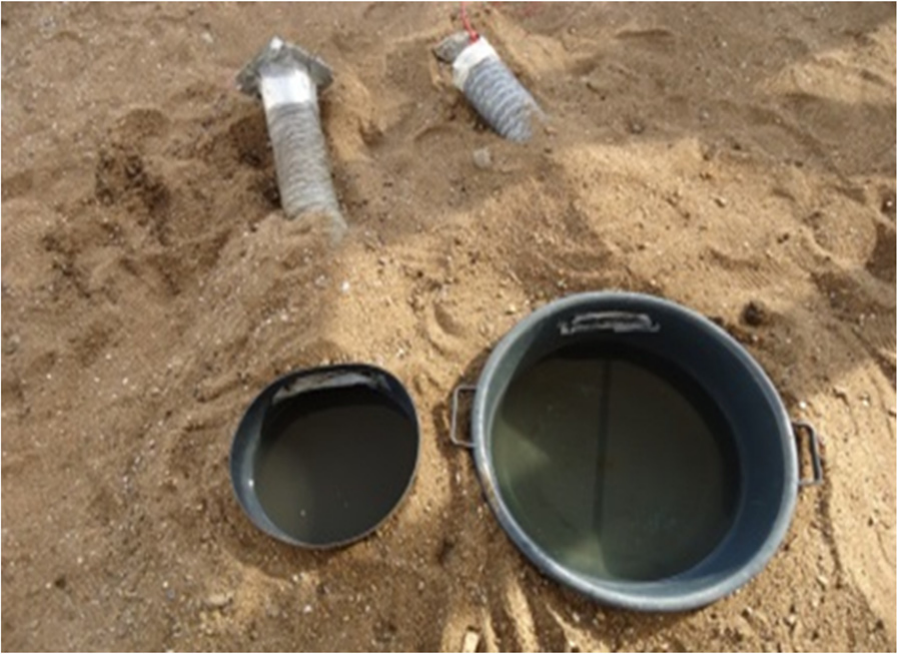
Comparison of the prototype OviART gravid trap with small basin (*left*) and improved trap with a basin twice the size of the prototype (*right*)

The prototype OviART gravid trap was made of a black round basin (height = 20 cm, diameter = 30 cm, volume = 8 l) to provide an aquatic habitat for oviposition, a 12 V 0.38 Amp fan powered by a 12 V battery that sucks air above the water surface through collapsible pipes, into a collection chamber, located on the side of the trap (Fig. 3) [12]. Two modifications to the prototype trap were tested: (i) a bigger basin containing double volume of the oviposition medium that increased surface area medium (height = 20 cm, diameter = 50 cm, volume = 16 l); and (ii) a stronger fan (12 V, 0.75A) fitted with the bigger basin. These two modified traps were compared consecutively with the prototype OviART gravid trap. The location of each treatment was randomly allocated to the opposite corners of the semi-field system each night. The number of mosquitoes collected in the traps and the number of eggs laid in their respective ponds was recorded nightly. The number of eggs was counted by filtering the water from the bowls through a filter paper (Fisher brand, QL 125) using a water suction vacuum pump. The bowls were rinsed with additional water and white filter papers were passed slowly along the edges of the bowls to detect any eggs that might have remained after rinsing.

#### Standardized field evaluation of six sampling devices targeted at gravid malaria vectors

Previously, we evaluated a range of sampling tools for trapping gravid females in a semi-field system [13]: (i) a Box gravid trap (also known as Reiter Cummings modified gravid trap, BioQuip, Rancho Dominguez, CA, USA), which is a commercially available and widely used trap for the collection of gravid *Culex* spp. and *Aedes* spp. mosquitoes [12, 16–18]; (ii) a square of e-nets around an artificial pond which we designed and built specifically for the collection of gravid females [13]; (iii) a transparent acetate sheet coated with insect glue (Oecos, UK) that floats on the water surface [11] of an artificial pond; (iv) an artificial pond that was treated on the water surface with an insect spray glue (Oecos, UK); (v) an artificial pond that was treated with 2.5 % detergent (Teepol LTD, Nairobi); and (vi) a cardboard rectangle (50 × 80 cm) covered with a transparent sticky foil (Barrettine, UK) that reflects light and has been shown to be highly attractive to gravid *Anopheles gambiae* (*s.s.*) even in the absence of water in semi-field system [13]. The specifications and their operations are described in detail in previous publications [13, 19]. All of them collected gravid females when released in a semi-field system and e-nets and detergent ponds were found to be particularly effective [13, 19]. This experiment was designed to investigate their performance in collecting wild mosquitoes in comparison to the commercial Box gravid trap under natural environmental conditions.

**Fig. 4:**
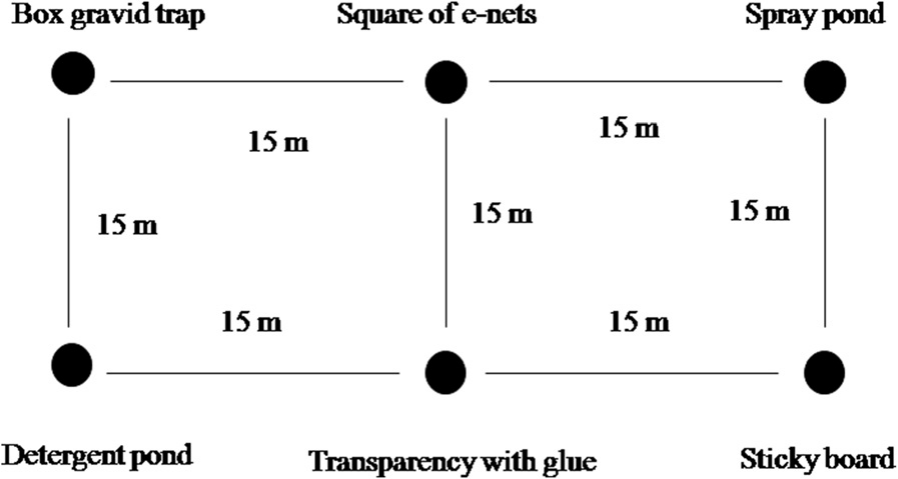
A graphical representation of the placement of the six catching devices tested under experimental field conditions

Three open field plots, each 50 × 35 m in area, were prepared by clearing all vegetation. Each plot was 200–300 m distant from the next. All three plots were approximately equidistant from human dwellings (70–100 m) and potential breeding sites (100–150 m). In each plot, six locations, 15 m apart (Fig. 4), were used to test the six different sampling devices. Once the devices were set up, they remained in their location for six nights before they were randomly allocated to another location within the plot. Traps were not allowed to be reallocated to the same position where it had been used previously. The experiments were repeated for a total of 24 nights. All sampling devices, except the cardboard covered with transparent sticky foil contained an artificial pond filled with 9 l of lake water. The water in the ponds was discarded after the 6th night and the basins were cleaned and the treatments assigned to another location within a plot. The treatments were set up between 15:00–17:00 h on the first days of each block and the e-nets and the traps were switched on daily at 18:00 h and switched off in the morning at 8.00 h.

#### Field comparison of the improved OviART gravid trap and a square of e-nets with the Box gravid trap

**Fig. 5:**
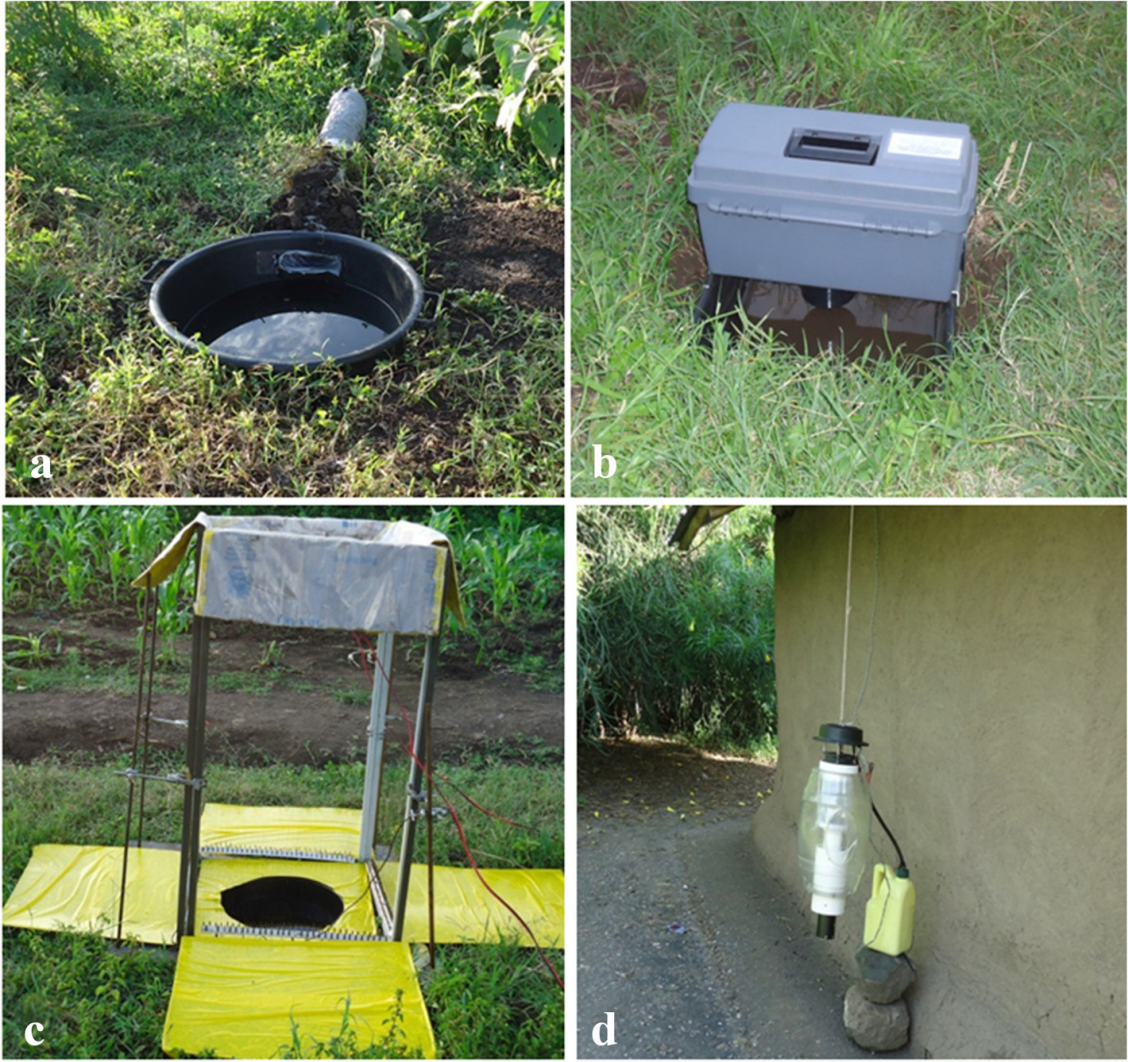
Traps set up for comparison under field conditions in Kombe village, western Kenya. **a** The OviART gravid trap. **b** The Box gravid trap. **c** Square of electrocuting nets around pond with yellow sticky boards to preserve electrocuted mosquitoes. **d** the MM-X trap fixed on outside of house

Verbal consent was sought from household heads of 12 homesteads requesting permission to trap mosquitoes on their land. The homesteads were grouped in three clusters of four homesteads. Homesteads within each cluster were 30–50 m apart and the minimum distance between clusters was 50 m. Within each homestead one trap location was selected 10–12 m away from an occupied house. Four different trap types were set up in parallel per night: Box gravid traps, squares of e-nets and OviART gravid traps for the collection of gravid mosquitoes and odour-baited Mosquito-Magnet-X traps (MM-X traps; American Biophysics Cooperation, RI, USA) for collecting host seeking mosquitoes (Fig. 5). The MM-X trap was baited with Nylon strips treated with the ‘Mbita blend’ [20] and CO_2_ generated from a mixture of yeast, sugar and water [21]. The MM-X trap was suspended from the edge of the roof of study houses so that the bottom of the trap was 15 cm above the ground [22], a position used routinely in the study area for mosquito monitoring [20, 22]. The mosquito collections with the MM-X trap served as a reference for estimating *Anopheles* species composition and densities in the study area since gravid collections were never routinely used in the field before. If the gravid traps did not trap gravid specimens of *Anopheles* spp. in the field, it would have been difficult to relate this to the absence of malaria vectors in the area in general or due to the ineffectiveness of the tools under field conditions.

A complete randomized block design was used to allocate trap locations per household cluster. Every cluster received four different traps and the traps were assigned randomly to the homesteads using a random number generator and rotated randomly across trapping locations within a cluster after every four nights of trapping. This was done for a total of 24 nights.

All traps were powered using 12 V batteries, except the Box gravid trap which used a 6 V battery. Nine litres of water freshly collected from the lake was used to fill the artificial ponds of the Box gravid trap and sixteen litres of water was added to the ponds with the square of electric nets and the OviART gravid trap. Experiments were run from 18.00 h until 08.00 h the following morning. Specimens were killed by subjecting them to -15 °C for 30 min. Mosquitoes electrocuted by the square of e-nets were carefully removed from the sticky collection boards surrounding the e-nets using forceps. The mosquitoes were morphologically identified as culicines and anophelines. The anopheline mosquitoes were further identified to the species level morphologically using keys developed by Gillies & Coetzee [23]. Members of the *An. gambiae* species complex were identified to species using PCR and gel electrophoresis [24]. All females in a good enough condition were dissected to examine their ovarial status. Freshly blood-fed and unfed females were categorised as non-gravid and females with matured eggs were categorised as gravid mosquitoes.

### Data analysis

The number of mosquitoes collected per trap type per site in the standardized field experiments was pooled for six consecutive sampling nights presenting one data point to reduce the number of zero catches and to account for the dependent nature of the samples. Therefore, a total of 24 nights were grouped into four weeks. For the consequent field study, the number of mosquitoes collected per trapping tool per site was pooled for the four consecutive sampling nights presenting one data point. Six weeks representing the sum of mosquitoes collected in four nights per week were analysed.

All count data were analysed using generalized linear mixed effects models (glmer-function) fit by the Laplace approximation with R statistical software version 2.14.2 including the contributing packages MASS, lme4, glht, multcomp (alpha = 0.05) [25]. The experimental night (semi-field tests) or week (field tests) and trap location were included in the models as random factors and the trap type was modelled as a fixed factor. The models were fitted using with a Poisson error and log link when the dependent variable was the number of mosquitoes and fitted with a binomial error and logit link when the dependent variable was a proportion. The excess variation between data points (overdispersion) that remained after adjustment for all other factors was adjusted by creating a random factor with a different level for each row of the data set. The prototype trap collections served as reference to which the other catches were compared in the experiments done in the semi-field and the Box gravid trap collections served as the reference in the two field experiments. The parameter estimates of the models were used to predict the mean counts or mean proportions per treatment and their 95 % confidence intervals (CI) by removing the intercept from the models [26]. Multiple comparisons of treatments were also calculated based on the model parameter estimates.

### Ethical approval

Ethical approval was obtained for feeding the mosquitoes on humans from the Kenya Medical Research Institute’s Ethical Review Committee (Protocol no. 422).

## Results

### Modifications of the prototype OviART gravid trap

Approximately twice [Rate Ratio (RR) 1.9, 95 % CI 1.1–3.4, *P* = 0.029] as many mosquitoes were collected with the modified OviART gravid trap with the larger pond compared with the prototype trap (Table 1). However, whilst hardly any eggs were found in the prototype trap, eggs were regularly found in the modified trap (Table 1) indicating either that the fan was not strong enough to collect all approaching mosquitoes, or not covering the entire surface area with its suction letting some of them lay and fly off, or some of the trapped mosquitoes got a chance to lay before they eventually got close enough to the suction to be collected. Consequently, a fan with a stronger suction was tested in the second experiment to test if more mosquitoes could be collected. However, using a stronger fan did not improve collections further. The mean numbers of mosquitoes collected using the OviART gravid trap with the larger basin and original fan and the OviART gravid trap with the larger basin and stronger fan were similar (Table 1); eggs were still found when a stronger suction fan was utilised. Despite these results, the stronger fan was selected for combination with a bigger pond for the final trap evaluated under natural field conditions since stronger air movements in the field were expected to interfere with the weaker suction.

**Table 1:** Association between treatments (pond size and strengths of suction fan) and catching efficiency of OviART gravid trap using a generalised linear mixed effects model

Treatment	Modelled mean per trap night (95 % CI)	Rate ratio (RR) (95 % CI)	*P*-value
Evaluation of pond size			
Adults collected by the traps			
Prototype trap	20.4 (13.5–30.9)	1	–
Trap with bigger basin and original fan	38.8 (26.0–57.9)	1.9 (1.1–3.4)	0.029
Eggs laid in traps			
Prototype trap	0.7 (0.1–3.2)	1	–
Trap with bigger basin and original fan	71.4 (19.4–262.7)	109.8 (25.3–76.3)	< 0.001
Evaluation of increased suction of fan			
Adults collected by the traps			
Prototype trap	26.3 (21.1–32.7)	1	–
Trap with bigger basin and stronger fan	36.2 (29.4–44.6)	1.4 (1.0–1.9)	0.037
Eggs laid in traps			
Prototype trap	6.5 (2.5–17.3)	1	–
Trap with bigger basin and stronger fan	124.3 (50.5–306.4)	19.0 (5.0–71.8)	< 0.001

### Standardized field evaluation of six sampling devices targeted at gravid malaria vectors

A total of 1,582 mosquitoes were collected with the six sampling devices over the 24 collection nights. The work was implemented at the beginning of the dry season, from the second week of June 2013 and overall density recorded was relatively low with an average of 4.3 specimens of *An. gambiae* (*s.l*.) mosquitoes collected by all the six devices over the three locations per trap night. Only 6.6 % (105) of the mosquitoes belonged to the *An. gambiae* species complex and the rest (1,477, 93.4 %) were culicines. Most of the mosquitoes were female (1,536, 96.6 %). The probability of collecting a specimen of *An. gambiae* (*s.l*.) was similar for the commercially available Box gravid trap and the pond with insect glue sprayed on the water surface whereas it was 3.3 (95 % CI 1.4–7.6, *P* = 0.006) times more likely to collect one with a square of e-nets (see Table 2 for all statistical analyses results). The sticky acetate sheet floating on the water surface performed poorly with five times less specimens of *Anopheles* spp. trapped than with the Box gravid trap and 16 times less than with the square of e-nets. The floating sticky acetate sheet was however very efficient in collecting culicine mosquitoes and performed as well as the square of e-nets and the pond spayed with insect glue on its surface collecting 3.7–5.5 times as many culicines as the Box gravid trap (Table 2). The detergent treated pond and the cardboard covered with a transparent sticky foil did not collect any *Anopheles* specimen under the open field condition and also performed poorly in collecting culicines (Table 2).

**Table 2:** Association between trap type and mosquito catch during the standardized field evaluation of six sampling devices using a generalised linear mixed effects model

Treatment	Modelled weekly mean (95 % CI)	Rate ratio (RR) (95 % CI)	*P*-value
*An. gambiae* (*s.l*.)^d^			
Box gravid trap	1.0 (0.3–2.7)	1^a^	–
E-nets	3.1 (1.2–8.0)	3.3 (1.4–7.6)^b^	0.006
Sticky water surface	0.9 (0.3–2.5)	0.9 (0.4–2.4)^a^	0.864
Floating sticky transparency	0.2 (0.1–0.9)	0.2 (0.1–0.9)^c^	0.029
Culicines			
Box gravid trap	5.6 (2.3–13.9)	1^a^	–
E-nets	21.2 (8.9–50.6)	3.8 (2.3–6.2)^b^	< 0.001
Sticky water surface	30.9 (13.0–73.5)	5.5 (3.4–9.0)^b^	< 0.001
Floating sticky transparency	20.8 (8.7–49.6)	3.7 (2.3–6.1)^b^	< 0.001
Detergent	4.3 (1.8–10.6)	0.8 (0.4–1.3)^a^	0.348
Sticky board	5.0 (2.0–12.4)	0.9 (0.5–1.5)^a^	0.693

^a,b,c^Multiple comparisons of treatments were calculated based on the model parameter estimates. Values sharing same letter were not statistically different (*P* > 0.05)

^d^Detergent and sticky boards did not collect *An. gambiae* (*s.l*.) mosquitoes. Therefore, they were not included in the model

Approximately 30 % (427) of females trapped were in good enough condition to be dissected to determine whether they were gravid or not. Overall, 88 % (95 % CI 82–94 %) of those were gravid. Of the 63 female *An. gambiae* (*s.l.*) that were successfully removed from the trapping tools 95 % (60) were *An. arabiensis* and 5 % (3) were *An. gambiae* (*s.s.*). No other species of *Anopheles* were found during these field experiments.

### Field comparison of the improved OviART gravid trap and a square of e-nets with the Box gravid trap

A total of 2,698 mosquitoes were collected in the field, 93 % (2,518) were female and 7 % (180) male. Most were culicines (85 %; 2,282), only 15 % (416) were *Anopheles* spp. Of the latter, 36 % (149) were *An. gambiae* (*s.l*.), namely 35 % (144) *An. arabiensis* and 1 % (5) *An. gambiae* (*s.s.*), 35 % *An. funestus* (*s.l*.) (145), 12 % *An. pharoensis* (50), 8 % *An. coustani* (33) and 9 % were not identified (39).

**Fig. 6:**
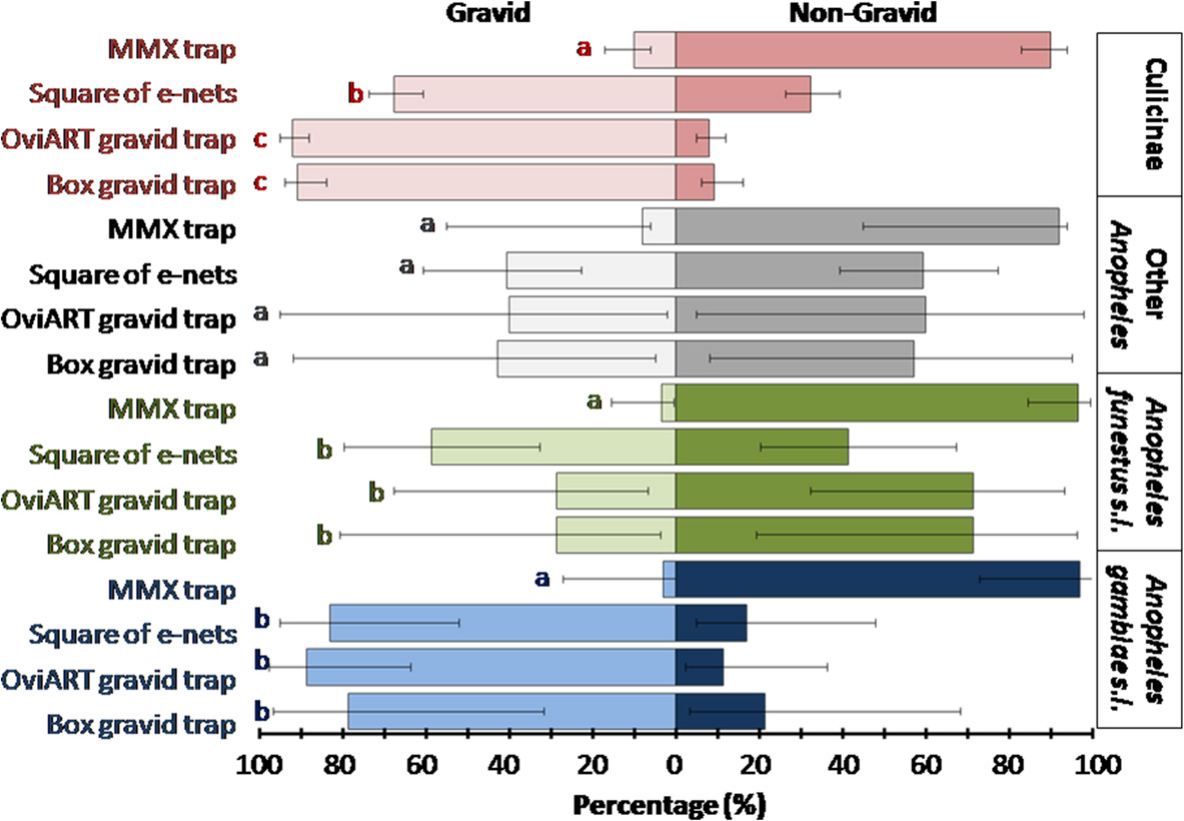
The percentage distribution of gravid versus non-gravid mosquitoes collected with four different trapping tools in the field based on generalized linear modelling. The vertical bars represent the 95 % confidence intervals. Multiple comparisons of treatments were calculated based on the model parameter estimates. Values sharing same letter (within a species group) were not statistically different (*P* > 0.05)

There was a high variability in the proportion of females that were gravid between species and sampling devices (Fig. 6). Two thirds (1,613/2,518) of the female mosquitoes collected were gravid. As expected the vast majority (97.3 %) of the gravid females were collected with the devices targeting them: the Box gravid trap, the OviART gravid trap and the square of e-nets. The MM-X trap with its artificial human odour targeted host-seeking mosquitoes and collected consequently for all species significantly (*P* < 0.05, Fig. 6) fewer gravid females than the other devices. Overall only 2.7 % of the total collection of the MM-X traps was gravid. Of all female mosquitoes trapped, 22 % were collected with the MM-X trap targeting host-seeking females.

The OviART gravid trap and the square of e-nets performed equally well under field conditions in collecting females of *An. gambiae* (*s.l*.) (Table 3). The OviART gravid trap collected a mean of 1.71 (95 % CI 0.87–3.40) *An. gambiae* (*s.l.*) females. It was three times (RR 3.3, 95 % CI 1.5–7.0, *P* = 0.003) more likely for a female to be trapped with the OviART gravid trap and two times (RR 2.3, 95 % CI 1.0–5.1, *P* = 0.041) with the square of e-nets than by the commercially available Box gravid trap.

**Table 3:** Association between trap type and mosquito catch during field evaluation using a generalised linear mixed effects model

	Box gravid trap (reference)	OviART gravid trap	Square of E-nets	MM-X trap
*Anopheles gambiae* (*s.l*.)				
Mean (95 % CI)	0.5 (0.2–1.2)	1.7 (0.9–3.4)	1.2 (0.6–2.5)	2.2 (1.1–4.3)
RR (95 % CI)	1^a^	3.3 (1.5–7.0)^b^	2.3 (1.0–5.1)^b^	4.2 (1.9–8.9)^b^
*P*-value	–	0.003	0.041	< 0.001
*Anopheles funestus* (*s.l*.)				
Mean (95 % CI)	0.3 (0.2–0.8)	0.7 (0.4–1.3)	1.4 (0.7–2.3)	4.2 (2.9–6.2)
RR (95 % CI)	1^a^	2.0 (0.8–5.4)^ab^	4.2 (1.7–10.3)^b^	12.6 (5.4–29.3)^c^
*P*-value	–	0.156	0.002	< 0.001
Other *Anopheles* spp*.*				
Mean (95 % CI)	0.2 (0.1–0.6)	0.2 (0.1–0.5)	2.7 (1.5–4.9)	1.0 (0.5–1.9)
RR (95 % CI)	1^a^	0.8 (0.2–3.3)^a^	11.8 (3.9–35.4)^b^	4.1 (1.3–13.0)^b^
*P*-value	–	0.722	< 0.001	0.016
Culicine species				
Mean (95 % CI)	16.0 (11.0–23.1)	29.2 (20.3–41.9)	31.8 (22.2–45.6)	19.6 (13.6–28.2)
RR (95 % CI)	1^a^	1.8 (1.26–2.7)^b^	2.0 (1.4–23.1)^b^	1.23 (0.8–1.8)^a^
*P-*value	–	0.002	< 0.001	0.284

^a,b,c^Multiple comparisons of treatments were calculated based on the model parameter estimates. Values sharing same letter were not statistically different (*P* > 0.05)

The MM-X trap collections indicate that *An. funestus* (*s.l*.) was the predominant species of *Anopheles* in the study area during the field trials (Table 3). However, this species composition was not reflected in the gravid trap collections, especially not with the Box gravid trap and the OviART gravid trap (Table 3). Furthermore, the specimen of the *An. funestus* group that were collected by these two traps were to a large proportion not gravid (Fig. 6). Similarly, other *Anopheles* spp. were underrepresented in these traps and largely not gravid (Table 3, Fig. 6). Squares of e-nets sampled specimen of the *Anopheles funestus* group and of other *Anopheles* spp. slightly more effectively, however also not specifically gravid females (Fig. 6).

## Discussion

The OviART gravid trap and the square of e-nets performed well under field conditions. The catching efficiency of *An. gambiae* (*s.l*.) for both devices was similar suggesting that most mosquitoes approaching a potential oviposition site, as measured by the e-nets [13, 19], were trapped by the suction fan of the OviART gravid trap when attempting to land on the water surface for oviposition [13]. The number of gravid *An. gambiae* (*s.l*.) collected with both tools was similar with the number of outdoor collected host-seeking females in the MM-X trap. This indicates that these tools can collect a representative number of wild gravid females of this vector species in the test environment where natural habitats were 200–400 m from the traps. Interestingly, both devices also collected a significantly larger number of culicines than the Box gravid trap.

The improved OviART gravid trap with a pond twice the size of the original prototype trap mimics natural *An. gambiae* (*s.l*.) larval habitats that are frequently man-made and found close to houses [27–29]. The increased catch size found with the bigger pond may be due to two reasons that probably interact; the increase in water surface probably increased the amount of water vapour and associated volatile odours that come off the habitat, acting as chemical attractants, and the light reflected from the water surface of the larger bowl might be more visible to a mosquito than a smaller one [30–32].

Of the devices used for sampling gravid females in the experimental field tests on icipe-TOC campus only the square of e-nets collected appreciable numbers of *An. gambiae* (*s.l.*) The square of e-nets surrounding an artificial pond was the most effective of the devices. This is consistent with the previous semi-field study [13]. Notably, the pond sprayed with insect glue on the water surface was very effective at collecting gravid culicines. The glue gave the water a sheen like oil and might appear like a surface film often associated with polluted water preferred by some *Culex* spp. for egg-laying [33–35]. The sticky transparent acetate sheet floating on the water surface also collected high numbers of culicines, as described previously [11] but performed poorly for collecting *Anopheles* spp. for which it was originally designed. All these devices collected more *Culex* spp. mosquitoes than the Box gravid trap. This might be in part due to the absence of chemical cues usually used in the Box gravid traps, i.e. hay infusions, to lure culicines that are attracted by volatile bacterial metabolites originating from decomposing organic matter [16-18]. In this study the culicine species were not further identified and the unexpected response might reflect a different species composition than usually targeted by Box gravid traps. Nevertheless, the high catching efficacy of the traps tested here should be further evaluated since they might be alternatives to the suction gravid traps used for collecting disease-transmitting culicines [16, 36]. However, the same sampling methods collected few, if any, *An. gambiae* (*s.l*.) and we hypothesise that this was due to visual deterrence caused by the conspicuous reflecting appearance caused by the glue or acetate sheet when used in small sized ponds. The ponds treated with 2.5 % detergent and the sticky cardboards without water caught no *Anopheles* spp. mosquitoes and only a low number of culicines. This result contrasts markedly with our semi-field study where these tools consistently collected high numbers of *An. gambiae* (*s.s.*) [13]. The reasons for these findings are not well understood but might be explained by the presence of alternative, more suitable/attractive choices in close vicinity.

Whilst the OviART gravid trap and square of e-nets worked well for the collection of *An. gambiae* (*s.l*.), they performed poorly for *An. funestus* (*s.l*.) and other *Anopheles* spp*.* This is not unexpected, based on the different nature of their larval habitats. *An. funestus* (*s.l*.), *An. pharoensis* and *An. coustani* are most frequently found in more permanent, natural water bodies with dense and tall vegetation and rarely in small (man-made) habitats without vegetation [27, 28]. Typical habitats for these species were abundant along the lake shore close to the households selected for the field study and explain their pre-dominance in the field collections whilst they were not collected in the standardised field trials on *icipe* campus further away from such habitats. The proportion of gravid mosquitoes collected was much lower for the *An. funestus* group, *An. pharoensis* and *An. coustani* females than for *An. gambiae* (*s.l*.) and culicines, suggesting that these were not approaching the artificial ponds of the trapping devices to lay eggs but either approached in response to the humidity or were trapped by chance whilst seeking for a blood-meal [37, 38]. Whatever the reason for being trapped, it is interesting that gravid traps might indeed be a tool for monitoring primary and secondary vector populations of all physiological stages outdoors.

The materials used to construct the OviART gravid trap are cheap and locally available. This new trap is cheaper and easier to construct than other gravid traps. However, since it is powered by a 12 V battery it remains vulnerable to theft unless it remains hidden or guarded. In addition, the trap could be damaged by animals and the battery is heavy to carry from one site to another. The square of e-nets is also a very effective tool for trapping gravid mosquitoes. However, it is impractical to use e-nets as an operational tool because of their less convenient portability, difficulty of construction and more liability to destruction from rain and animal interference in the field. Nevertheless, it is an effective novel research tool that could be used to study important events such as oviposition cues (repellence, attraction) and distribution of gravid females in space.

## Conclusion

The modified OviART gravid trap with a water surface area of 0.07 m^2^ (706.5 cm^2^) was found to be an effective tool for trapping gravid mosquitoes both under semi-field and field conditions. The relative ease of handling this trap provides opportunity to develop it further in a monitoring and possibly even control tool targeting gravid malaria vectors outdoors. Furthermore, the square of e-nets presents an alternative sampling tool that collects gravid mosquitoes as they approach and could be used as a research tool to investigate abundance and dispersal of gravid vectors as well as to validate other outdoor sampling tools.

The current field evaluation was done in an area where natural habitats were in close but not immediate vicinity (200–400 m) so that the traps located close to houses intercepted females on their way from the host to the oviposition sites. In this scenario it is unlikely that the gravid females perceived visual or chemical cues from the natural habitats a minimum of 200 m away. Furthermore, a recent study has shown that trap’s efficacy can be enhanced if an attractant oviposition cue for anophelines is added to the water [39]. However, further field testing of this novel trap must be done in different eco-epidemiological settings with different mosquito population densities and varying distances to natural habitats to validate the presented findings and to provide recommendations for its use as a surveillance tool.
